# Association of vitamin B12 deficiency with metformin use in patients with type 2 diabetes treated in the largest tertiary care hospital in Qatar

**DOI:** 10.5339/qmj.2021.39

**Published:** 2021-09-09

**Authors:** Fahmi Yousef Khan, Abdelmonem B. Yousif, Aasir Suliman, Ahmed Osman Saleh, Mohamed Magdi, Awni Alshurafa, Ebtihal Abdelmoneim Hassan, Ahmed Ghazy, Omar K. Salameh, Ahmed Abdallah

**Affiliations:** ^1^Department of Medicine, Hamad General Hospital, Doha, Qatar E-mail: fakhanqal@yahoo.co.uk; ^2^Department of Pharmacy, Hamad General Hospital, Doha, Qatar; ^3^Department of Accident & Emergency, Hamad General Hospital, Doha, Qatar

**Keywords:** Vitamin B12 deficiency, type 2 diabetes mellitus, metformin, sulfonylurea compounds

## Abstract

Background: Data on the effect of metformin on serum vitamin B12 (VitB12) level in patients with type 2 diabetes mellitus (T2DM) in Qatar are limited; therefore, we aimed to assess the prevalence of VitB12 deficiency and its related factors among patients with tbl2DM treated with metformin at Hamad General Hospital in Doha, Qatar, from January 1, 2017, to December 31, 2017.

Methods: This cross-sectional analytical study involved patients with tbl2DM aged ≥ 18 years who used metformin for at least 3 months. The serum VitB12 was quantified on a chemiluminescent enzyme immunoassay analyzer using Cobas e 801 module, Roche, and VitB12 deficiency was defined as serum VitB12 level of ≤ 145 pmol/L. All data were obtained from the patients’ electronic medical records.

Results: The study recruited 3124 eligible patients with tbl2DM. The overall prevalence of metformin-associated VitB12 deficiency was 30.7% [95% confidence of interval, 0.290–0.323]. A significant difference exists in the median VitB12 levels between the VitB12-normal and VitB12-deficient groups [129 vs. 286; p < 0.001]. Compared with the VitB12-normal group, the VitB12-deficient group had higher mean body mass index (BMI) (*p* < 0.001) and consumed higher doses of metformin (*p* = 0.001). They also more often used sulfonylurea (*p* = 0.004), dipeptidyl peptidase-4 inhibitor (*p* < 0.001), thiazolidinediones (*p* < 0.001), glucagon-like peptide 1 [GLP-1] receptor agonists (*p* < 0.001), alpha-glucosidase inhibitor (*p* < 0.001), and H2 blocker/proton pump inhibitors [PPI] (*p* < 0.001) than the VitB12-normal group. Moreover, the VitB12-normal group consumed more calcium supplements (*p* < 0.001) than the VitB12-deficient group. In the multivariate analysis, independent risk factors for metformin-associated VitB12 deficiency in patients with tbl2DM include high daily dose of metformin >2000 mg, male gender, high BMI, smoking, sulfonylurea, dipeptidyl peptidase-4 inhibitor, H2 blockers/PPI, low fasting blood glucose, and low hemoglobin.

Conclusion: This study showed a high prevalence of VitB12 deficiency in patients with tbl2DM taking metformin and a significant negative correlation between the daily dose of metformin and serum VitB12 level. Therefore, regular screening for serum VitB12 is necessary in patients with tbl2DM on metformin treatment, especially those who have the abovementioned risk factors.

## Introduction

Metformin is an important drug used worldwide as a first-line treatment for type 2 diabetes mellitus (T2DM) as recommended by American and European diabetic associations.^1–3^ Metformin exerts its antidiabetic effect by increasing insulin sensitivity in the liver and decreasing glucose production. However, it has side effects such as lactic acidosis, diarrhea, nausea, vomiting, flatulence, and decreased serum level of vitamin B12 (VitB12).^1–4^

VitB12 malabsorption associated with metformin use was first speculated in early 1971. Since then, several reports from different centers have supported this assumption. VitB12 is an essential cofactor for converting homocysteine to methionine and for regenerating folate, leading to DNA synthesis and myelin sheath formation. Thus, enzymatic defects resulting from VitB12 deficiency lead to an accumulation of methylmalonic acid and homocysteine, which causes various hematological, gastrointestinal, and neuropsychiatric disorders.^4,5^

There is a lack of information on the prevalence of metformin-associated VitB12 deficiency in patients with tbl2DM in Qatar. This is because the only study conducted prior to ours did not show a significant difference in serum VitB12 levels between metformin and nonmetformin users.^6^ Thus, this study aimed 1) to determine VitB12 deficiency prevalence in patients with tbl2DM who used metformin from January 1, 2017, to December 31, 2017; 2) to assess the relationship between VitB12 deficiency and utilization of metformin; and 3) to identify predictors of VitB12 deficiency among our patients in our setting.

## Patients And Methods

### Design, population, and setting

This cross-sectional analytical study was conducted at Hamad General Hospital (HGH), Doha, Qatar. It involved patients with tbl2DM treated with metformin from January 1, 2017, to December 31, 2017.

### Patient identification and source of data

All patients were identified from the records of the pharmacy department at HGH, and their details were obtained from electronic medical records (Cerner system). Patient enrollment for the study was based on the following criteria: patients with tbl2DM, age ≥ 18 years, daily use of metformin for at least 3 months, and VitB12 levels are available in the electronic records. Patients who did not meet the abovementioned inclusion criteria were excluded. Moreover, vegetarians; pregnant women; patients recently diagnosed with tbl2DM (less than 3 months), pernicious anemia, and type 1 diabetes mellitus; patients who underwent gastrectomy, colectomy, and/or patients who had inflammatory bowel disease were excluded. Patients previously receiving injections of VitB12 and patients with serious medical conditions such as sepsis, severe infection, malignancy, heart failure, and liver cirrhosis were also excluded.

### Definitions

The serum VitB12 was quantified on a chemiluminescent enzyme immunoassay analyzer using Cobas e 801 module, Roche. VitB12 deficiency was defined as a serum VitB12 level of ≤ 145 pmol/L, and anemia was considered if the hemoglobin level was < 13 g/dl in men and < 12 g/dl in women. Patients were diagnosed with hypertension if their systolic blood pressure was ≥ 140 mmHg and/or diastolic blood pressure was ≥ 90 mmHg or if they were receiving any antihypertensive medication. Diabetic nephropathy was defined as glomerular filtration rate (GFR) of less than 60 mL/min/1.73 m^2^

### Data collection

The following data were retrieved from the electronic records of the patients: demographic information, BMI, DM duration, duration of metformin use, daily dose of metformin, underlying medical conditions (e.g., hypertension, diabetes, and diabetic nephropathy), alcohol use, smoking cigarettes, concomitant usage of drugs (e.g., insulin, sulfonylureas, dipeptidyl peptidase-4 inhibitor, thiazolidinediones, glucagon-like peptide 1 (GLP-1) receptor agonists, alpha-glucosidase inhibitor, histamine H2 blockers/proton pump inhibitors [PPI], calcium supplements, and multivitamins), and investigation results (e.g., total cholesterol, low-density lipoprotein (LDL)-cholesterol, high-density lipoprotein (HDL) cholesterol, triglycerides (TGs), glycosylated hemoglobin levels, fasting blood glucose, mean corpuscular volume (MCV), hemoglobin, serum creatinine, VitB12 level, and estimated GFR [eGFR].

### Sample size

The prevalence of metformin-associated VitB12 deficiency in patients with tbl2DM varies between 9% and 43%.^1,7^ Assuming a prevalence “P” of 40% and based on an absolute precision error (d) of 5% and a type I error (α) of 5%, the calculated sample size was 368. However, for an accurate estimation of the population parameters, 3,124 patients were included, representing all cases that met the inclusion criteria during the study period. Therefore, a complete enumeration has been performed.

### Data analysis

Descriptive statistics of qualitative and quantitative data were expressed as frequency along with percentage and mean ( ± SD). Shapiro–Wilk test was used to assess the normality of the variables. Variables with non-Gaussian distribution were transformed using log transformation. Thereafter, the normality of the transformed variables was re-evaluated, and only a few variables could not be transformed to follow the Gaussian distribution. Pearson's chi-square tests were used to test the differences in the proportion of categorical variables to determine presence of a significant relationship between metformin use and VitB12 deficiency. Independent t-tests were used to evaluate the difference between the means of two continuous variables that were normally distributed, while Mann–Whitney U-test was used to compare differences between two independent groups with the assumption that the data did not follow a normal distribution. A Pearson correlation analysis was performed to examine the linear relationship between serum VitB12 and metformin use. Multiple logistic regression analysis was performed to assess the independent predictive effect of the variables as a risk for VitB12 deficiency. Univariate analysis was performed to determine the probable risk factors for VitB12 deficiency. Variables with *p* < 0.05 values in the univariate analysis were entered in the multiple logistic regression to identify the independent risk factors for VitB12 deficiency at *p* < 0.05. Data were analyzed using the SPSS software (v 25; IBM Corp, Armonk, NY, USA).

### Ethical approval

Ethical approval was obtained from Medical Research Committee (MRC) at Hamad Medical Corporation (Proposal no. MRC-01-18-066).

## Results

**Figure 1.**
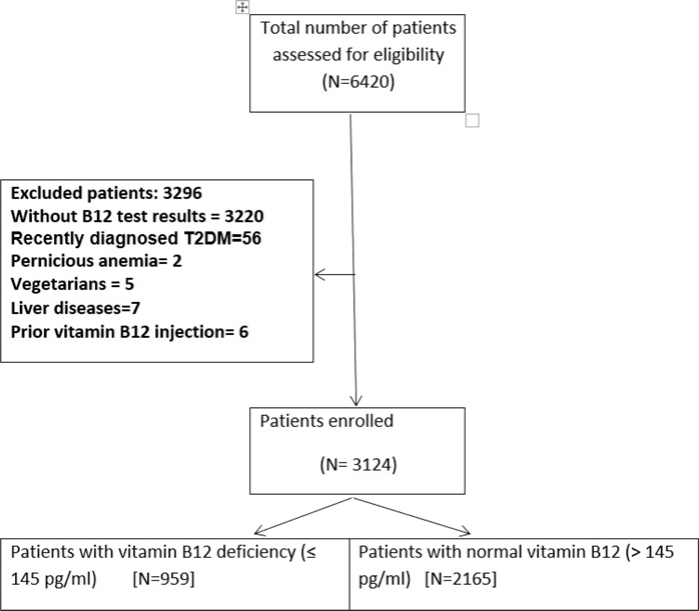
Flow chart of patient recruitment

A total of 6420 patients were identified during the study period, out of which 3124 eligible patients with tbl2DM were recruited in this study (Figure 1). The mean age of the patients was 56.6 ± 10.2 years, with 72% being men and 28% being women. The demographic and clinical details of the patients are described in Table 1. Of the 3124 patients, 959 (30.7%) were found to have serum VitB12 deficiency [95% confidence interval (CI) (0.278–0.336)].

### Comparison of demographic and clinical characteristics influencing VitB12 level in patients with tbl2DM treated with metformin

No significant differences were noted in age, nationality, DM duration, duration of metformin use, diabetic complication (hypertension and diabetic nephropathy), antidiabetic medications (insulin, sodium-glucose cotransporter-2 inhibitors), multivitamins, mean total cholesterol, mean LDL-cholesterol, median HDL-cholesterol, mean glycosylated hemoglobin level, MCV, mean serum creatinine, and eGFR between the VitB12-normal and VitB12-deficient groups (Table 1).

A significantly higher percentage of the VitB12-deficient patients were men (*p* = 0.003), and the mean BMI in this group was high compared with that in the VitB12-normal group (*p* < 0.001). The VitB12-deficient group also consumed significantly higher doses of metformin (*p* = 0.001) and had higher rates of anemia (*p* = 0.001), smoking (*p* = 0.019), and alcohol consumption (*p* < 0.001) than the other group. Moreover, the VitB12-deficient group more often used sulfonylurea (*p* = 0.004), dipeptidyl peptidase-4 inhibitor (*p* < 0.001), thiazolidinediones (*p* < 0.001), GLP-1 receptor agonists (*p* < 0.001), alpha-glucosidase inhibitor (*p* < 0.001), and H2 blocker/PPI (*p* < 0.001) than the VitB12-normal group. The mean hemoglobin (*p* < 0.001) and mean fasting blood glucose (FBG) (*p* < 0.001) were significantly low, while the median of TG was significantly high (*p* < 0.001) in the VitB12-deficient group compared with that in the other group. In addition, the VitB12-normal group consumed more calcium supplements [404(42.17%) vs. 86(3.97%); *p* < 0.001 (Table 1)] than the VitB12-deficient group. Table 1 describes the differences between the VitB12-deficient group and VitB12-normal group in relation to different demographic and clinical aspects.

### Correlation between metformin use and VitB12 deficiency

Table 2 explains the relationship of the serum level of VitB12, daily dose of metformin, and duration of metformin use. A significant negative association was noted between the daily metformin dosage and serum VitB12 levels (r = − 0.32, *p* = 0.01), while no significant correlation was observed between the duration of metformin use and serum VitB12 levels (r = 0.02, *p* = 0.10).

### Multivariate logistic regression analysis of factors associated with VitB12 deficiency

Table 3 demonstrates the association of various risk factors with VitB12 deficiency. In the multivariate analysis, and after adjusting for various confounders such as gender, nationality, BMI, daily dose of metformin, hypertension, alcohol use, smoking, sulfonylurea, dipeptidyl peptidase-4 inhibitor, thiazolidinediones, GLP-1 receptor agonists, alpha-glucosidase inhibitor, calcium supplement, and TG level, independent risk factors identified for metformin-associated VitB12 deficiency in patients with tbl2DM included a high daily dose of metformin >2000 mg, male gender, high BMI, smoking, sulfonylurea, dipeptidyl peptidase-4 inhibitor, H2 blockers/PPI, fasting blood glucose, and low hemoglobin. Calcium supplements showed a significant negative regression coefficient ( − 2.810), indicating a protective effect.

## Discussion

Although several studies have shown that the use of metformin has an important effect on VitB12 levels in patients with tbl2DM, no published recommendations have required regular screening tests for VitB12 deficiency in these patients. In its most recent report (2019), the American Diabetes Association recommended regular screening for VitB12 level in metformin-treated patients with tbl2DM who had anemia or peripheral neuropathies.^8^ To the best of our knowledge, this study is the largest report in Qatar that investigated the effect of metformin use on serum VitB12 among patients with tbl2DM.

Several studies have reported metformin-associated VitB12 deficiency with various frequencies depending on the cut-off values used to diagnose VitB12 deficiency.^1–4,6,7,9^ The overall prevalence of VitB12 deficiency in our study was 30.7%, which falls within the global range of 9%–43%.^1,7^ In addition, this study showed male gender predominance regardless of their nationality, which can be explained by the fact that Qatar and other Gulf countries have a large working community composed mainly of men.

In the bivariate analysis (Table 1), we found a significant difference in the serum levels of VitB12 between VitB12-normal and VitB12-deficient groups [286 pmol/L vs 192 pmol/L; *p* < 0.001], which is in line with several reports worldwide.^1–4,7,9–17^ Our results are also consistent with those of several studies,^1,3,7,9–19^ which showed a significant relationship between metformin dosage and serum VitB12 level with a variation in metformin dosage cut-off values. Saqer et al., and Alsaeed et al.,^18,19^ found that a daily metformin dose of ≥ 1000 mg was associated with VitB12 deficiency, while Kim et al.,^9^ found that VitB12 deficiency was significantly associated with daily metformin doses of ≥ 1500 mg. However, several reports, including ours,^1,7,17,20,21^ observed that VitB12 deficiency was associated with daily metformin doses higher than 2000 mg. Moreover, several studies have illustrated a significant negative correlation between metformin dose and VitB12 serum level,^3,10,13,16,17,20,22^ which is consistent with our finding.

The exact mechanism of metformin-associated VitB12 deficiency is not fully understood. Among the mechanisms proposed, metformin is thought to induce malabsorption of VitB12-intrinsic factor complex in the terminal ileum by affecting the calcium-dependent membrane action.^15,23^ Furthermore, some authors suggested that metformin-associated VitB12 malabsorption is due to increased bacterial overgrowth or modification of the gut microbiota as a result of a delay in the absorption of glucose.^24^

Many studies have investigated the correlation between the duration of metformin use and serum VitB12 level; however, the obtained data showed a contradiction. Several authors found a negative correlation between the duration of metformin use and VitB12 levels.^1–3,13–15,18^ Conversely, the duration of metformin use was not significantly associated with serum VitB12 level in our study (Table 2) and in other studies.^16,17,20,21,25,26^ This contradiction could be attributed to the differences in the criteria used for diagnosing VitB12 deficiency, patient characteristics, and/or metformin dosage.

The effect of the concomitant use of H2 blocker/PPI with metformin on serum VitB12 level is inconclusive, as results of various reports are contradictory. Many reports have shown that VitB12 level is not associated with concomitant use of H2 blocker/PPI,^1,6,14,18,23^ while our findings (Table 1) and results of two reports^16,27^ showed that the coadministration of H2 blocker/PPI and metformin was associated with a significant reduction in serum VitB12 levels. The reduced acidity, attributed to the prolonged use of H2 blocker/PPI, may lead to VitB12 malabsorption by two mechanisms that is, inadequate release of VitB12 from food proteins, which affects the absorption of VitB12 in the terminal ileum, and excessive growth of bacteria in the intestine.^24^

In agreement with Kand et al.,^28^ we found a low serum level of VitB12 when sulfonylurea was used in combination with metformin. Furthermore, coadministration of metformin with dipeptidyl peptidase-4 inhibitor, thiazolidinedione, alpha-glucosidase inhibitor, and GLP-1 receptor agonist was also significantly associated with low serum VitB12 levels (Table 1). The reason for this finding is unclear, so prospective studies are needed to confirm and to investigate its mechanism.

In this study, we estimated the MCV and hemoglobin levels in the VitB12-normal and VitB12-deficient groups to evaluate the influence of VitB12 deficiency on these hematological parameters. Like other reports,^8,14,16,23,25^ we found a significant difference in the number of anemic patients and the mean hemoglobin levels between the two groups (Table 1). There was a high prevalence of anemia among VitB12-deficient group compared with the VitB12-normal group. However, in contrast with some reports,^14,16,25,29^ we did not find a significant difference in the mean MCV between these two groups, which is consistent with few reports in the literature.^14,17,28^ Although VitB12-related anemia is commonly thought to have a higher MCV, previous studies have shown that up to 30% of VitB12-related disorders have normal MCVs.^14^ However, this finding can be attributed to the coexistence of other types of anemia, which mask the effect of VitB12 deficiency on this parameter. Therefore, in this study, metformin-associated anemia is most likely a multifactorial clinical disease that requires careful work to distinguish VitB12-related anemia from anemia of other causes.

Interestingly, a high number of patients received calcium supplements in the VitB12-normal group compared with the VitB12-deficient group (Table 1), which is consistent with the report of Bauman et al.,^30^ and supports the hypothesis that metformin causes malabsorption of the VitB12-intrinsic factor complex in the ileum by affecting the calcium-dependent membrane action.

Some studies have suggested that daily multivitamin supplementation may protect against VitB12 deficiency in patients with tbl2DM who received metformin.^31,32^ By contrast, we did not find a significant difference in the number of patients with multivitamins in both groups (Table 1), which makes the protective role of this supplement questionable. Conversely, we were unable to assess the effect of VitB12 supplements on our patients because either the supplements were not prescribed for most patients for unknown reasons or the patients were not taking the prescribed supplements regularly; therefore, we excluded this variable.

Many studies have analyzed independent risk factors for metformin-associated VitB12 deficiency. Koh et al.,^14^ found anemia, a high daily dose of metformin, and a long duration of metformin therapy as important independent risk factors, while Kang et al.,^28^ reported that the use of sulfonylurea in combination with metformin is a significant independent risk factor for VitB12 deficiency. Alharbi et al.,^1^ revealed that female gender, weekly VitB12 intake, high daily metformin dose, and medium-high MCV were risk factors for metformin-associated VitB12 deficiency, while Aroda et al.,^33^ found that years of total metformin exposure were the only significant predictor of VitB12 deficiency. In our study, calcium supplements, interestingly, showed a significant negative regression coefficient, which supports the protective role of these supplements against VitB12 deficiency in metformin-treated patients with tbl2DM. Furthermore, independent risk factors for VitB12 deficiency in metformin-treated patients with tbl2DM include daily dose of metformin >2000 mg, male gender, high BMI, smoking, low hemoglobin, high fasting blood glucose, and concomitant use of sulfonylurea, dipeptidyl peptidase-4 inhibitor, and H2 blockers/PPI (Table 3).

This study has several limitations. First, it was a retrospective analysis and therefore relied on secondary data that did not allow adequate assessment of some variables, such as treatment duration, disease duration, and effect of VitB12 supplements on peripheral neuropathy. Second, patients’ electronic records did not include the serum levels of other markers of VitB12 deficiency, such as methylmalonic acid and homocysteine; therefore, we missed the opportunity to increase awareness of recognizing B12 deficiency at its early stage. Third, as it was a hospital-based study, the results cannot be generalized. Despite these limitations, the strength of our study lies in the large number of patients enrolled.

## Conclusions

This study showed a high prevalence of VitB12 deficiency in metformin-treated patients with tbl2DM. A daily dose of metformin more than 2000 mg, male gender, high mean BMI, smoking, low hemoglobin, and concomitant use of sulfonylurea, dipeptidyl peptidase-4 inhibitor, and H2 blockers/PPI were independent risk factors for metformin-associated VitB12 deficiency in Patients with tbl2DM. Therefore, regular screening for serum VitB12 is necessary in patients with tbl2DM on metformin treatment, especially those who have the abovementioned risk factors.

### Conference presentation

This article was presented (oral presentation) at the Fourth Qatar Diabetes, Endocrinology, and Metabolic Conference (QDEM-4), which was held at Sheraton Hotel, Doha-Qatar from February 13 to 15, 2020.

### Availability of data and materials

All data are fully available without restriction and from the corresponding author on reasonable request. However, restrictions apply to the availability of these data, which are used under license for the current study.

### Authors contribution

**Khan FY** wrote the proposal, analyzed the data, and wrote the final manuscript; **Yusif AB** proposed the idea and review the literature; **Sulaiman A** aided in the data collection and research proposal writing; **Saleh AO** aided in the data collection and data entry; **Magdi M** aided in data collection and the first revision of the manuscript; **Alshurafa A** helped in data collection and proposed the idea; **Hassan EA** helped in data collection and revised the manuscript; **Ahmed G** proposed the idea and aided in the data collection; **Salameh OK** aided in the data collection and data entry; **Abdullah A** aided in the data collection and preparation and proposed the idea. All authors read the manuscript and agree to publication.

### Ethical approval

Obtained from medical research committee at Hamad medical corporation. (proposal number # MRC-01-18-066)

### Funding

The present study is investigator-initiated research. The authors have not received specific funding.

### Consent to participate

Not applicable.

### Competing interests

The authors declare no conflict of interests.

## Figures and Tables

**Table 1:** Demographic and clinical characteristics of the patients with and without vitamin B12 deficiency

**Variables**	**Total**	**Normal B12**	**B12 deficiency**	***p*** **value**

Number of patients	3124	2165 (69.30)	959 (30.70)	

**Demographic and clinical characteristics**				

Age (mean ± SD years)	56.59 ± 10.18	56.4 ± 10.13	57.03 ± 10.28	0.1136^ a^

Male (%)	2236 (71.57)	1515 (69.98)	721 (75.18)	0.003^**c**^

Qatari	215 (6.88)	138 (6.37)	77 (8.03)	0.092

BMI (mean ± SD)	29.99 ± 6.04	29.73 ± 5.91	30.57 ± 6.26	< 0.001^ a^

Diabetic duration [median years (IQR)]	7(5–11)	7(5–11)	8(5–11)	0.176^ b^

Daily dose of metformin mean ± SD mg)	1842.19 ± 379.05	1827.25 ± 400.35	1875.91 ± 323.46	0.001^ a^

< 1000 mg	26 (0.83)	20 (0.92)	6 (0.63)	0.007^**c**^

1000–2000 mg	2986 (95.58)	2053 (94.83)	933 (97.29)	

>2000 mg	112 (3.59)	92 (4.25)	20 (2.09)	

Hypertension (%)	2110 (67.56)	1481 (68.44)	629 (65.59)	0.117^**c**^

Diabetic nephropathy (%)	262 (8.39)	190 (8.78)	72 (7.51)	0.238^**c**^

Anemia (%)	1309 (41.91)	751 (34.7)	558 (58.19)	< 0.001^**c**^

Alcohol user (%)	292 (9.35)	169 (7.81)	123 (12.83)	< 0.001^**c**^

Smoker (%)	844 (27.02)	558 (25.77)	286 (29.82)	0.019^**c**^

**Medications**				

Insulin (%)	773 (24.74)	522 (24.11)	251 (26.17)	0.218^**c**^

Sulfonylurea (%)	1155 (36.97)	765 (35.33)	390 (40.67)	0.004^**c**^

Dipeptidyl peptidase-4 inhibitor(%)	1146 (36.7)	749 (34.61)	397 (41.4)	< 0.001^**c**^

Thiazolidinediones (%)	293 (9.38)	175 (8.09)	118 (12.3)	< 0.001^**c**^

GLP-1 receptor agonists (%)	253 (8.1)	134 (6.19)	119 (12.41)	< 0.001^**c**^

SGLT2 inhibitors (%)	885 (28.33)	601 (27.76)	284 (29.61)	0.289^**c**^

Alpha-glucosidase inhibitor (%)	445 (14.24)	246 (11.36)	199 (20.75)	< 0.001^**c**^

Multivitamin (%)	1288 (41.23)	883 (40.79)	405 (42.23)	0.449^**c**^

Calcium supplement (%)	490 (15.69)	404 (42.17)	86 (3.97)	< 0.001^**c**^

H2 blocker/PPI (%)	1247 (39.93)	638 (29.48)	609 (63.5)	< 0.001^**c**^

**Investigation results**				

Fasting blood glucose (mmol/L)	7.75 ± 2.49	7.87 ± 2.65	7.48 ± 2.06	< 0.001^ a^

Creatinine (mircomol/L)	78.38 ± 24.07	78.35 ± 23.82	78.44 ± 24.66	0.918^ a^

eGFR (mL/min/1.73 m^2^)	2862 (91.61)	1976 (91.27)	886 (92.39)	0.299^ a^

TC (mmol/L)	4.15 ± 1.11	4.16 ± 1.11	4.13 ± 1.10	0.529^ a^

TG [median(IQR)] (mmol/L)	1.48 (1.1–2.0)	1.4 (1.1–1.9)	1.6 (1.2–2.0)	< 0.001^ b^

HDL-cholestrol [median(IQR)] (mmol/L)	1.10 (0.9–1.4)	1.10 (0.9–1.4)	1.10 (0.9–1.6)	0.363^ b^

LDL-cholesterol (mmol/L)	2.08 ± 0.92	2.09 ± 0.89	2.07 ± 0.97	0.556^ a^

HbA1c (%)	7.46 ± 1.52	7.48 ± 1.57	7.42 ± 1.42	0.341^ a^

Urea (mmol/L)	4.99 ± 1.81	4.99 ± 1.81	5.00 ± 1.81	0.801^ a^

Hemoglobin (g/dl)	12.99 ± 1.78	13.19 ± 1.76	12.54 ± 1.74	< 0.001^ a^

MCV (fL)	84.15 ± 8.63	84.19 ± 7.86	84.05 ± 8.15	0.678^ a^

Macrocytosis (MCV>100 fl)	11 (0.35)	1 (0.05)	10 (1.04)	< 0.001^ c^

Vitamin B12 [median(IQR)] (pg/ml)	232(155–333)	286 (227–391)	129 (120–155)	< 0.001^ b^


^a^ 2-tailed t-test; ^b^ Independent-samples median test; ^c^ 2-tailed Pearson chi-square test

**BMI**, body mass index; **eGFR**, estimated glomerular filtration rate; **GLP-1**, glucagon-like peptide 1; **HbA1c,** glycosylated hemoglobin; **HDL**, high-density lipoprotein; **IQR**, interquartile range; **LDL**, low-density lipoprotein; **MCV**, mean corpuscular volume; **PPI**, proton pump inhibitors; **SD**, standard deviation; **SGLT2**, sodium-glucose cotransporter-2; **TC**, total cholesterol; **TG**, triglyceride

**Table 2:** Correlation between the daily dose of metformin, duration of metformin use, and serum vitamin B12 level

**Correlation between the dose of Metformin and Vitamin B12 level**	

Correlation coefficient (r)	− 0.32*

*p* value	0.01

Number of patients (N)	3124

*A significant negative linear relationship exists between the daily dose of metformin and serum vitamin B12 level	

**Correlation between the duration of Metformin Use and Vitamin B12 level**	

Correlation coefficient (r)	0.02

*p* value	0.10

Number of patients (N)	3124

No significant linear relationship exists between the daily duration of metformin use and vitamin B12 level	


**Table 3:** Results of the multivariate analysis for potential independent risk factors of metformin-associated vitamin B12 deficiency in patients with tbl2DM

**Variables**	**Adjusted Odds Ratio [95% CI]**	***p*** **value**

Men	2.24(1.77–2.84)	< 0.001

BMI	1.04(1.02–1.05)	< 0.001

Daily dose of metformin >2000 mg	1.96(0.35–2.59)	0.001

Smoker	0.75(0.60–0.95)	0.015

Sulfonylurea	1.25(1.03–1.53)	0.025

Dipeptidyl peptidase-4 inhibitor	1.32(1.09–1.61)	0.005

Calcium supplement	1.6(0.74–1.81)	< 0.001

H2 blocker/PPI	1.79(1.47–2.18)	< 0.001

Low FBS (mmol/L)	0.93(0.89–0.97)	< 0.001

Low hemoglobin (g/dl)	0.88(0.83–0.93)	< 0.001


**BMI**, body mass index; **FBS**, fasting blood sugar**; PPI**, proton pump inhibitors;
